# Prevalence of DNA-HPV in Male Sexual Partners of HPV-Infected Women and Concordance of Viral Types in Infected Couples

**DOI:** 10.1371/journal.pone.0040988

**Published:** 2012-07-17

**Authors:** Maria Gabrielle de Lima Rocha, Fabio Lopes Faria, Leonor Gonçalves, Maria do Carmo M. Souza, Paula Ávila Fernandes, Ana Paula Fernandes

**Affiliations:** 1 Department of Clinical and Toxicology Analyses, Federal University of Minas Gerais, Belo Horizonte, Brazil; 2 Central-South Polyclinic – Center of Sexually Transmitted Diseases from the Central-South District of the City Hall of Belo Horizonte, Belo Horizonte, Brazil; IPO, Inst Port Oncology, Portugal

## Abstract

Investigation of HPV infection in men remains important due to its association with genital warts and anorectal cancer, as well as to the role men play in HPV transmission to their female sexual partners. Asymptomatic men (n = 43), whose sexual partners had presented cervical HPV infection, were enrolled in this study. Among the 43 men, 23 had their female partner included and tested for HPV-DNA, totaling 23 couples. HPV-DNA was detected by PCR. Type specific PCR to detect HPV 16, 18, 31, 33, 45 and 6/11 was performed. At least one type of HPV was detected in 86.0% (37/43) of the male patients and more than one HPV type was identified in 39.5% (17/43) of the samples, including high and low risk HPV. HPV-16 proved to be the most prevalent viral type in both male and female samples. Concordance of at least one viral type was observed in 56.5% (13/23) of the couples. Among couples that have shown concordance of viral types, 84.6% (11/13) of the men had the same high risk viral type presented by the female sexual partner. These data suggest that HPV infected men is an important reservoir, contributing to a higher transmission to women and maintenance of infection, and consequently, a higher risk of developing cervical cancer. HPV vaccination in men will protect not only them but will also have implications for their sexual partners.

## Introduction

Genital human papillomavirus (HPV, family Papilomaviridae, genus Alpha-papilomavirus) infection is one of the most common sexually transmitted diseases. It is estimated that the prevalence of HPV-infection is 20% of all men, reaching 70% in some age groups, especially among individuals between 15 and 24 years of age. Although HPV infection in men may be associated with low mortality and morbidity, investigation remains important due to its association with genital warts, penile cancer, anorectal cancer and oropharyngeal cancer as well as to the role men play in HPV transmission to their female sexual partners [1]–[3].

Transmission can occur easily between sexual partners, and in many cases, multiple transmission events may take place with a couple without being detected [4]–[7]. However, as compared to HPV infection in women little is known about the natural history of HPV infection in men.

Compared to cervical cancer, penile cancer is a relatively rare disease and generally occurs late in life. Approximately 40% of penile carcinomas can be attributed to infection with high risk HPV, whose presence is related mainly to basaloid and warty histological subtypes [7], [8]–[10]. Globally, HPV infection accounts for an estimated 530,000 cervical cancer cases (∼270,000 deaths) annually, with the majority (86% of cases, 88% of deaths) occurring in developing countries. Approximately 90% of anal cancers and a smaller subset (<50%) of other cancers (oropharyngeal, penile, vaginal, vulvar) are also attributed to HPV. In total, HPV accounts for 5.2% of the worldwide cancer burden. HPVs 16 and 18 are responsible for 70% of cervical cancer cases and, especially HPV 16, for a large proportion of other cancers. Prophylactic vaccination targeting these genotypes is therefore expected to have a major impact on the burden of cervical cancer as well as that of other HPV-related cancers [3]. Even though anal cancer is more prevalent in females than in males, the risk of anal cancer is higher in men who have sex with men than the risk of cervical cancer in women [2].

The HPV type concordance between sex partners has been addressed in previous studies [11]–[14]. Marked variations in HPV type concordance are evident, which may be explained by differences in the number of HPV types studied, the different methods used for penile sampling, and the population studied. Moreover, some studies have demonstrated that type-specific concordance may well be related to the amount of viral DNA [15].

Several lines of evidence have suggested that the sexual behavior of males can contribute to the risk of cervical cancer in their sexual partners [5], [7], [10], [16]. Therefore, strategies to limit HPV infections in men may result in health gains. HPV vaccination in men will protect not only them but will also have implications for their sexual partners.

The prevalence of HPV infection detected in male partners of women who have received a positive diagnosis for this virus, and/or cervical intraepithelial neoplasia or squamous carcinoma, varies between 23% and 73% [4], [9], [11]–[14]. However, few studies have evaluated the concordance of HPV types between couples or the prevalence of HPV infection in sexual partners of women with cervical lesions caused by an HPV infection in the Brazilian population [9], [12]–[14], [17].

Therefore, this study aimed to detect the prevalence of DNA-HPV in the male partners of HPV-infected women and to assess the concordance of viral types in infected couples.

## Material and Methods

### Studied Population

**Figure 1:**
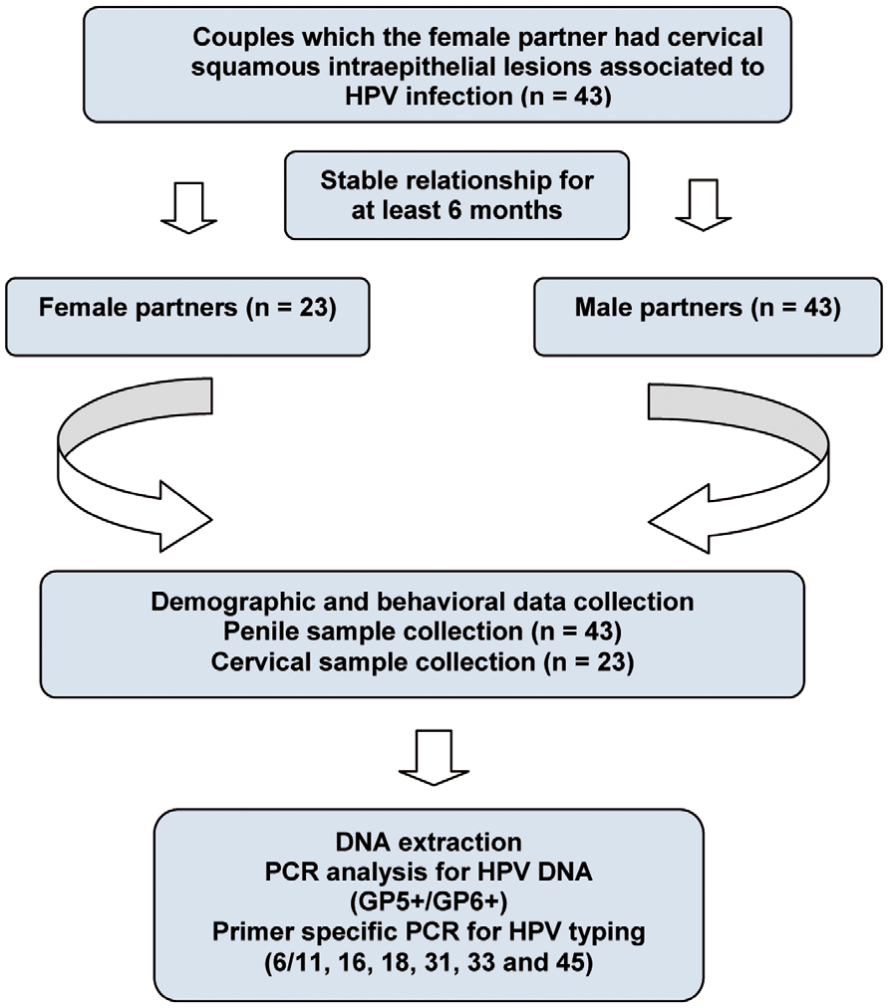
Study flow diagram.

Based on the cytological or histopathological diagnosis of cervical squamous intraepithelial lesions associated to HPV infection of the female partner, couples were invited to participate of this study. Of the invited couples, all the 43 male partners attended to diagnostic evaluation and were included, while only 23 women attended for a new sample collection and had their cervical samples tested for HPV-DNA, totaling 23 couples (Figure 1). Besides the diagnosis of cervical squamous intraepithelial lesions associated to HPV infection of the female partner, the maintenance of steady relationships for at least 6 months was also adopted as an inclusion criterion for couples. All patients attended to the public health center for sexually transmitted diseases in the city of Belo Horizonte, Minas Gerais, Brazil (Central-South Polyclinic – Center for training in Sexually Transmitted Diseases from the Central-South District of the City Hall of Belo Horizonte, Minas Gerais, Brazil) for diagnostic evaluation.

### Ethics Statement

The study protocol was approved by the Ethics Committee of Research of Federal University of Minas Gerais (protocol 0088.0410203-09). Information concerning the research project was provided to all participants, and all signed a free and informed consent form approved by the institutional ethics committee of the Federal University of Minas Gerais (UFMG).

### Sample Collection

The penile samples were collected using the DNA Citoliq system (Digene, Brazil). Material was brushed from the glans and prepuce internal surfaces, including the sulcus and the corona, and placed in transport tubes. Cells were then suspended in a preservative liquid, which allowed them to be used for both liquid-based cytology and DNA extraction (UCM – Universal Collecting Medium, Digene, Brazil). The cytopathological changes observed in these samples had been previously reported [18]. The cervical samples were collected by gynecologists using the DNA Citoliq system (Digene, Brazil).

### Molecular Analysis

DNA extraction was carried out in aliquots obtained from the 1 mL liquid medium samples (200 µL for cervical and 400 µL penile samples). The molecular analysis was performed in DNA obtained using proteinase K, phenol:chloroform extraction, and isopropanol DNA precipitation. All samples were submitted to PCR, using the oligonucleotides PC03 and PC04, which amplified a 120 pb sequence of a human β-globulin gene, so as to create an internal control to verify the integrity and quality of the extracted DNA [19]. The GP5+/GP6+ oligonucleotides were used to detect HPV-DNA [20]. Positive samples which had been previously tested for HPV infection were included in each set of reactions as positive controls. Type-specific PCR was also performed to detect HPV 16, 18, 31**,** 33**,** 45, and 6/11, using primers and conditions presented in Table 1. Each sample was tested in triplicate, and negative samples were tested in different DNA concentrations to confirm results. Reactions were prepared with 0.05 mM of each dNTP (GIBCO-BRL, USA); 1 U of Taq polymerase (Phoneutria, MG, Brazil), 1.5 mM of MgCl_2_, and the specific oligonucleotides in a final volume of 10.0 µL. PCR were performed in MJ PT100 thermocyclers (MJ Research, MA, USA) and consisted of 35 cycles of 1 min. at 94°C for denaturing, 1 min. at 55°C for annealing, and 1 min. at 72°C for extension to amplify the human β-globulin gene fragment. To amplify the HPV-DNA, a “touch-down” PCR condition was used, which consisted of 5 initial cycles of 1 min. at 95°C, 1 min. at 45°C, and 1 min. at 72°C, followed by 35 cycles of 1 min. at 95°, 1 min. at 45°C, and 1 min. at 72°C. Negative and positive controls were included within the settings for each reaction. PCR products were analyzed in silver stained 6% polyacrylamide gel electrophoresis. Oligonucleotides and PCR conditions for genotyping HPV through PCR are listed in Table 1.

**Table 1:** Oligonucleotides and PCR annealing temperature used for identification and genotyping HPV.

PCR (pb)	Oligonucleotides (pb)	Annealing temperature (°C)
**β-globulin**	5′ACA CAA CTG TGT TCA CTA GC 3′	55
PC03/PC04 (100)^1^	5′CAA CTT CAT CCA CGT TCA CC 3′	
**HPV-DNA**	5′TTT GTT ACT GTG GTA GAT ACT AC 3′	45/42
GP5+/GP6+ (150)^2^	5′GAA AAA TAA ACT GTA AAT CAT ATT C 3′	touch down
**HPV 16** (152)^3^	5′TGC TAG TGC TTA TGC AGC AA 3′	57
	5′ATT TAC TGC AAC ATT GGT AC 3′	
**HPV 18** (216)^3^	5′AAG GAT GCT GCA CCG GCT GA 3′	65
	5′CAC GCA CAC GCT TGG CAG GT 3′	
**HPV 31** (514)^3^	5′ATG GTG ATG TAC ACA ACA CC 3′	54
	5′GTA GTT GCA GGA CAA CTG AC 3′	
**HPV 33** (455)^3^	5′ATG ATA GAT GAT GTA ACG CC 3′	57/55
	5′GCA CAC TCC ATG CGT ATC AG 3′	touch down
**HPV 45** (296)^3^	5′TTT GTT GGC ATA ATC AGT TGT TTG 3′	59
	5′CAA AAC GAT ATG TAT CCA CCA AAC T 3′	
**HPV 6/11** (301)^3^	5′TAC ACT GCT GGA CAA CAT GC 3′	68/65
	5′GTG CGC AGA TGG GAC ACA C 3′	touch down

1 SAIKI, 1988.

2 RODA HUSMAN, 1995.

3 GRCE, 1997 and HUANG, 2004.

### Statistical Analysis

Statistical significance for prevalence of different HPV types between age groups or gender was tested by using Proportion Test Z and considered significant when p<0.05.

## Results

Table 2 shows the demographic characteristics and sexual behavior among the 43 men studied. Of these, 25 men were between 18 and 30 years of age, while the others (n = 18) were between 31 and 60 years of age. The 43 men and 21 women were negative for genital warts and were not vaccinated against HPV infection. Among the female partners, 20 presented low grade and 3 high grade squamous intraepithelial lesions. None of the asymptomatic men presented other STD. The majority of men reported monogamous relationships, which was defined as a stable sexual partner for more than 6 months. The average age on initiating sexual life was 15.3 years old and average number of sexual partners on the last year was 1.4.

**Table 2:** Demographic characteristics and sexual behavior among 43 asymptomatic men studied.

Variable	Number of patients
Age (years)	
18–30	25
>30	18
Monogamous (stable sexual partner for more than 6 months)	35
Weekly sexual frequency	
1–4	39
>4	4
Current smokers	
yes	12
no	31
Condom use	
yes	6
no	37
Anal intercourse	
yes	8
no	35
Circumcision status	
yes	3
no	40

The prevalence of DNA-HPV among the 43 penile samples, as detected by PCR, is shown on table 3. Among the male patients analyzed, 86.0% (37/43) presented at least one type of HPV, although in only 51.2% (22/43) patients was DNA-HPV detected by GP5+/GP6+ oligonucleotides. In 39.5% (17/43) of the samples, more than one HPV type was detected. Of the 17 men displaying multiple viral types, 15 had low and high risk HPV concomitantly. Among the 43 patients, 33% (14/43) presented only high risk HPV and 18.6% (8/43) only low risk HPV. In 2 patients, the HPV type could not be determined. Six penile samples were negative in all PCR for HPV detection, including PCR using GP5+/GP6+ oligonucleotides. Among these samples, 3 were collected in man whose female partner was also included in the study. As shown in table 4, the prevalence of the different HPV types did not differ by comparing men 18–30 years old and those aged more than 30 years, except for HPV 31 (p = 0.036).

**Table 3:** Prevalence of DNA-HPV among the 43 penile samples as detected by PCR.

HPV type	n (%)	Viral types (n)
HPV (GP5+/GP6+)	22 (51.2)	
At least 1 viral type	37 (86.0)	
Only high risk HPV	14 (33.0)	
Only low risk HPV	8 (18.6)	
More than 1 viral type	17 (39.5)	
High and Low risk HPV	15 (34.8)	6/11+16 (4)
		6/11+31 (4)
		6/11+18 (3)
		6/11+16+18 (1)
		6/11+16+31 (1)
		6/11+16+31+33 (1)
		6/11+16+31+45 (1)
High risk HPV	2 (4.6)	31+33 (1)
		16+18+31 (1)

**Table 4:** Prevalence of HPV viral types in men according to age.

HPV type	n (%)	P Value
HPV 6/11	21(48.8)	
18–30 years	14/25 (56)	0.271
>30 years	7/18 (39)	
HPV 16	15 (34.9)	
18–30 years	10/25 (40)	0.416
>30 years	5/18 (28)	
HPV 18	08 (20.9)	
18–30 years	4/25 (16)	0.617
>30 years	4/18 (22)	
HPV 31	12 (27.9)	
18–30 years	10/25 (40)	0.036
>30 years	2/18 (11)	
HPV 33	02 (4.7)	
18–30 years	1/25 (4)	0.763
>30 years	1/18 (6)	
HPV 45	01 (2.3)	
18–30 years	1/25 (4)	0.391
>30 years	0/18	
HPV Non typed	2/43 (4.7)	
18–30 years	0/25	0.090
>30 years	2/18 (11)	
Negative DNA-HPV	6/43 (13.9)	
18–30 years	2/25 (8)	0.190
>30 years	4/18 (22)	

Cervical and penile samples collected in 23 couples were also submitted to HPV genotyping. The prevalence of viral types in these samples is shown in Table 5. The detection of HPV by GP5+/GP6+ oligonucleotides in 95.7% (22/23) of the female samples was higher than in 52.1% (12/23) of male samples (p = 0.0008). No significant difference (p = 0.39) was observed for prevalence of low risk HPV between female (47.8%) and male samples (35%). HPV 16 proved to be the most prevalent viral type in both male and female samples (p = 0.817). HPV 31 was two times more prevalent in male, as compared to female samples (p = 0.043), while HPV 18 was the second most prevalent viral type among female samples (p = 0.056).

The concordance of viral types between couples is shown in Table 6. Concordance of at least one viral type was observed in 13 (56.5%) of the 23 couples. Concordance of all viral types was observed in only one case (4.3%). Among couples that have shown concordance of viral types, 85% (11/13) of the men had the same high risk viral type presented by the female sexual partner.

**Table 5:** HPV types prevalence as detected by PCR in cervical and penile samples of 23 couples.

Viral Type	Women (%)	Men (%)	p
HPV (GP5+/GP6+)	22/23 (95.7)	12/23 (52.1)	0.0008
HPV 6/11	11/23 (47.8)	7/20 (35.0)	0.396
HPV 16	13/23 (56.5)	12/20 (60.0)	0.817
HPV 18	11/23 (47.8)	4/20 (20.0)	0.056
HPV 31	3/23 (13.0)	8/20 (40.0)	0.043
HPV 33	2/23 (8.7)	2/20 (10.0)	0.884
HPV 45	1/23 (4.3)	1/20 (5.0)	0.913

* Six penile samples were negative in all PCR for HPV detection. Among these samples, 3 were collected in man whose female partner was also included in the study.

* Statistical significance for prevalence of different HPV types between age groups was tested by using Proportion Test Z.

**Table 6:** Concordance of viral types between sexual partners (23 couples).

		Types HPV	
Concordance	Number of couples	Man	Woman
**Total**	**1 (4.3%)**	**16**	**16**
**Partial**	**12 (52.2%)**	**16,** **6/11**	**16**, 18, **6/11**
		**31**, 33	18, **31**, 6/11
		**6/11**	18, **6/11**
		**18**	**18**, 31, 6/11
		**16**, 31	**16**, 18
		**16**	**16**, 31
		**16**, 18, 31, 45, 6/11	**16**
		**16**, 31, 33, **6/11**	**16**, 18, **6/11**
		16, **6/11**	18, **6/11**
		**16**, 31, 6/11	**16**
		**18**, 31	16, **18**,33
		**16**	**16**,18,33
**Absent**	**10 (43.47%)**	Negative	16, 6/11
		16	45, 6/11
		16	18
		HPV non typed	16, 6/11
		16, 18, 31	6/11
		31, 6/11	16, 18
		HPV non typed	16, 6/11
		6/11	16
		Negative	16
		Negative	18

## Discussion

HPV infection was prevalent in 86% of the men in the present study. Previous data of other Brazilian studies [12]–[14] reported the prevalence of HPV infection among sexual partners of women with cervical squamous lesion varying to 23% to 70%. The increased prevalence observed in the present study (86%) may be due to sampling and/or the method used for HPV detection. The small sample analyzed and selection bias due to the different inclusion criteria adopted in different studies may have also affected results. Sampling at multiple penile sites, when lesions are not visible, apparently increases the sensitivity of the HPV infection detection. High rates of detection are generally reported in samples collected from glans, coronas, prepuces, and penile bodies, as compared to those collected in scrota, urethra, urine, and semen [5]. Although increased prevalence in men whose sexual partners had been diagnosed with HPV infection is indeed expected, a high prevalence of HPV infection among Brazilian sexual partners of women with and without cervical cancer, as compared to those detected in other countries (Spain, Colombia, Thailand, Philippines, USA and Mexico), has been also reported in prior literature [9], [21].

At least one HPV type appeared in 37 men (86%), although in only 22 of these (51.2%) DNA-HPV was detected by means of GP5+/GP6+ oligonucleotides. By contrast, the detection of HPV by GP5+/GP6+ oligonucleotides in female samples, as compared to male samples, was higher, suggesting that a higher cellular representation may be present in cervical samples, in turn improving the chances of detecting HPV. In men, several factors may affect the detection of DNA-HPV, such as the absence of a delimited lesion, the excess of cornea squamous, and lower viral loads. Negative results when consensus oligonucleotides are used in positive samples, as detected by specific oligonucleotides, have been reported in prior studies [22]–[24]. One possible explanation for this discrepancy may be that during the integration of the viral genome, a portion of the L1 region, where the annealing of consensus oligonucleotides occurs, may be deleted, whereas the region where the pairing of type-specific primers occurs remains unaffected.

In the present study, among the 43 male subjects, 33% presented only high risk HPV. Moreover, multiple infections of high risk and low risk HPV (39.5%) were also frequently present, which is in agreement with prior reports [12]. None of the male partners presented any visible lesions, although it has been reported that penile lesions are more frequent in partners of high risk HPV carriers [25]. However, the presence of subclinical penile lesions cannot be ruled out in these sexual partners. Penile lesions, the majority of which are subclinical, may be present in 45% to 68% of the sexual partners of HPV positive women [13], [26], [27].

By comparing the concordance of viral types between couples, it has been observed that 56.5% (13/23) of the couples shared at least one viral type, while 47.8% (11/23) of the male subjects shared at least one high risk viral type with their partners. Studies evaluating the HPV infection in men who are sexual partners of women with intraepithelial squamous lesions and/or HPV have shown that 13% to 63.2% of partners are infected by the same HPV viral type [11], [12], [14], [28], suggesting that concordance is more frequent than expected by chance [4]. On the other hand, the lack of concordance in a proportion of couples may be explained by differences in the time required for clearance of HPV infection in men and women and the time of relationship between couples [4], [7]. Moreover, HPV 16 and 18 infections seem to be more persistent than other viral types and thus differences between viral types regarding clearance time may also affect concordance between couples [29].

In addition, the lack of concordance of HPV types in 43.5% of couples may be explained by the fact that the transmission of HPV infection occurs mainly at the beginning of sexual life and is associated with immunity [30], [31]. Immune responses may influence the viral load, the alternation of viral types, the individual propagation of HPV types, and therefore, concordance between partners with long-term relationships. Nonetheless, the concordance observed (56.5%) is similar to those reported in other studies [28], [32], [33] and supports the evidence that HPV infected men has significant role on the maintenance of the transmission chain.

The prevalence of viral types in partners of infected women varies in different studies, presenting rates of 3.5% to 59% for HPV 16, from 3.5% to 6.7% for HPV 18, and from 3.5% to 8.5% for HPV 31 [9], [11], [13]. While the prevalence of high risk HPV 16, 33, and 45 were similar among men and women, in the present study, HPV 31 was the second most prevalent viral type in men and proved to be two times more prevalent in men than in women (p = 0.043). These findings have epidemiological implications, considering the recommendation of HPV vaccination for both men [34] and women, as already established in some countries.

The low number of patients and couples studied, as well as an insufficient monitoring of the duration and clearance of the HPV infection among couples, creates certain limitations to the present study. It would be also interesting to genotype all HPV positive samples for additional viral types. Thus, further investigation is needed to better elucidate these facts. Although similar studies have been conducted in different countries, it is still important to investigate the different epidemiological aspects regarding HPV infection in men, attempting to reproduce and confirm results. Moreover, these findings, including those reported herein, may help to better comprehend men’s role in the transmission of this infection, to guide adoption and to follow up of control measures, such as vaccination and the alterations in epidemiological patterns of this significant STD.
